# Supplementary figures and images for: Development and assessment of an immobilized bacterial alliance that efficiently degrades tylosin in wastewater
Source: PLoS One. 2024 May 31;19(5):e0304113. doi: 10.1371/journal.pone.0304113 (PMC11142594; doi:10.1371/journal.pone.0304113)

**Fig. S1.** Oxford cup results of TYL-A1 and TYL-B2. 1, 2, 3 for the experimental group, 4 for the blank control.

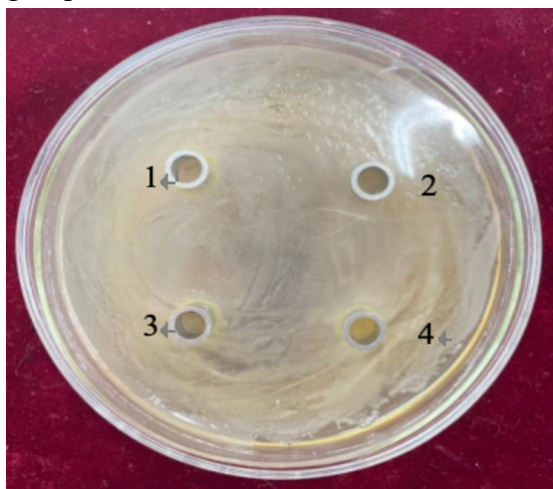

Supplement: S1 Fig — 1, 2, 3 for the experimental group, 4 for the blank control. (PDF) [file pone.0304113.s001.pdf]

**Fig. S2.** The characteristics of the beads with different AC contents, a)-e) were 0.5%, 1%, 1.5%, 2%, and 2.5%, respectively.

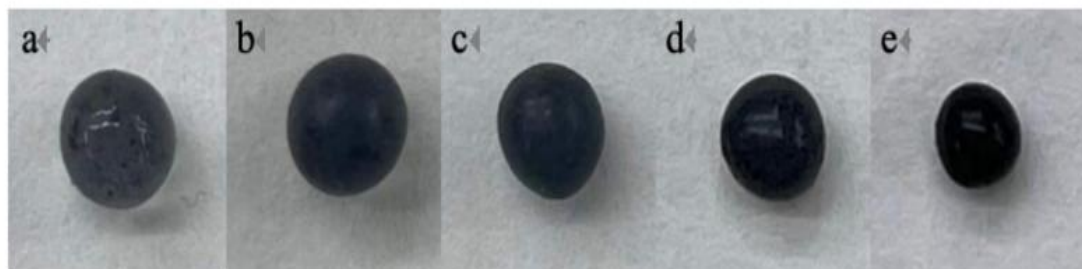

Supplement: S2 Fig — The characteristics of the beads with different AC contents, a)-e) were 0.5%, 1%, 1.5%, 2%, and 2.5%, respectively. (PDF) [file pone.0304113.s002.pdf]
