# Supplementary material for: Development and assessment of an immobilized bacterial alliance that efficiently degrades tylosin in wastewater
Source: PLoS One. 2024 May 31;19(5):e0304113. doi: 10.1371/journal.pone.0304113 (PMC11142594; doi:10.1371/journal.pone.0304113)
Supplement: Attachment — Submitted filename: Response to Reviewers.pdf [file pone.0304113.s003.pdf]

Response Letter

Manuscript ID: PONE-D-23-42913

Title: Development and assessment of an immobilized bacterial alliance that efficiently degrades tylosin in wastewater

Dear Editor,

Thank you very much for giving us an opportunity to submit a revised draft of the manuscript titled “ Development and assessment of an immobilized bacterial alliance that efficiently degrades tylosin in wastewater” for publication in the PLOS ONE. We highly appreciate your good self and the reviewers for dedicating time and efforts to providing feedback on our manuscript and we are grateful for the insightful comments and valuable suggestions for the improvement of our article. Their suggestion are of course worth learning. We have incorporated all the suggestions made by the reviewers. We have made extensive corrections to our previous draft and the modified parts are marked in red in the article. The following are responses to the reviewer's comments one by one.

Response:

Thank you for pointing out these problems in manuscript. We have made changes based on your valuable comments.

Reviewers’ comments

Reviewer 1

Comment : Line 18: “PVA-SA-AC” should be defined upon first use

Response: Thank you for your nice suggestion,We have re-added the definition in the article, where "PVA-SA-AC," polyvinyl alcohol-sodium alginate-activated carbon, is an immobilized biofiller.

Comment : Lines 74-77: The poor sentence construction, please check

Response: Thank you very much for your suggestion.The poor sentence construction has been changed.

Comment : Line 79: what is TSM?

Response: Thanks to your comment.TSM in the article, which stands for thifensulfuron methyl, is a sulfonylurea herbicide with high efficiency and low toxicity.

Comment : Lines 86-87: “from an agricultural environment.” What is the agricultural environment? Please be more specific when formulating the purpose of the work.

Response: Thank you very much for your valuable advice.The agri-environment, which is the sum of the elements of the surrounding environment, including agricultural land, water use, atmosphere, organisms, etc., which is the subject of crop or agricultural production, has been further described in the article.

Comment : Line 121: “spresd” please check

Response: Thank you very much for your suggestion.The “spresd” has been changed to “spread”

Comment : Line 147: “0.22 mm membrane” please check the units are correct.

Response: Thank you for your correction.The “0.22 mm membrane” has been changed to “0.22 um membrane”

Comment : Line 186: “in wastewater TYL” change to "in wastewater containing TYL"

Response: Thank you for pointing out the problems in the manuscript.We have replaced “in wastewater TYL” with"in wastewater containing TYL"

Comment : Lines 186-187: “standing wastewater and supernatant were” please give more detail what is standing wastewater and supernatant?

Response: Thank you very much for pointing out the problem.Sorry for the confusion caused by our misrepresentation. We are very sorry, and we have corrected it in the original article.

Comment : Line 188: “wastewater containing TYL” how much TYL is contained in wastewater?

Response: Thank you for your comment, the concentration of TYL in its wastewater is 75mg/L.

Comment : Line 189: “NH+ 4−N” please change to “NH4+-N”. Please use the same format for other nitrate-N and phosphate-P

Response: Thank you very much for your comment, the formatting of nitrate-nitrogen and phosphate-phosphorus in the article has been corrected.

Comment : Line 246: what are CTC and OTC?

Response: Thank you very much for pointing out the problem in the manuscript, where CTC stands for Chlortetracycline and OTC stands for oxytetracycline.

Comment : Line 261: “++++ softened…” please check, should be +++

Response: Thank you for your advice. The "+ + + + " in the article has been changed

to" +++"

Comment : Line 266: What does the word “concretization” mean here?

Response: Thank you very much for your advice, where "concretisation" means alliance, bacteria concretization, stands for the TYL-A1 and TYL-B1 consortia.

Comment : Line 279: “in contrast to inefficient degradation by free bacteria” degradation by free bacteria was not inefficient

Response: Thank you for your comment. The description may not be clear, because the degradation of immobilised beads in the first 2d accelerated the TYL degradation due to the synergistic effect of biodegradation and physical adsorption, and the degradation efficiency was higher than that of free bacterial consortium and blank beads ($p < 0.01$), and the description about this place in the text has been corrected

Comment : Line 293: "a" and "b" are not shown on images

Response: Thank you very much for your valuable advice， they have been changed over in the manuscript

Comment : Line 315: “represent SEM of three experiments” please define SEM here

Response: Thank you for your comment.SEM stands for scanning electron microscope, which is a means of observation intermediate between transmission electron microscopy and optical microscopy, and has been redefined in the manuscript by adding

Comment : Line 315: “wastewater from aquaculture” Wastewater from aquaculture or agriculture? please clarify

Response: Thank you for your comment.In the manuscript it was agricultural

wastewater, which has been changed.

Comment : Line 373: "Degradation efficiency was reduced, but it can be recycled." the meaning is not clear, please rephrase

Response: Thank you very much for your suggestion to rephrase this sentence, which has been revisited in the manuscript in which this issue has been re-addressed. Although immobilised strains have low degradation efficiency, immobilised beads can be recycled to save energy.

Comment : Line 376: "initial increase was attributed to TYL and immobilized carriers" please state your thesis more clearly. How could TYL and the immobilized carrier contribute to the increase in COD?

Response: Thank you for your comment. In the presence of TYL and immobilized carriers, too much of the composite carbon source leads to an increase in the concentration of organic matter and a corresponding increase in the value of COD; where too much of the composite carbon source also leads to the metabolic rate of microorganisms being accelerated, the organic matter in the wastewater will be decomposed by the organisms rapidly, generating more COD and leading to an increase in the COD content. This section has been revisited in the manuscript.

Comment : Lines 376-377: "whereas the decrease was attributed to degradation of TYL" what was the COD equivalent of TYL contained in wastewater?

Response: The COD equivalent of TYL in wastewater is 10847.6mg/L

Comment : Line 380: "probably due to bacteria utilizing P" Why was there a difference compared to the control?

Response: Thank you very much for pointing out the problem. First, the experimental group P is used by the bacteria as a nutrient for growth, and second, the bacteria and immobilized goblet cells act to further degrade the P, so the total P in the experimental group is significantly lower than the control group.

Comment : Lines 380-382: not a logical explanation. First, ammonia levels generally do not increase during denitrification. Secondly, this is an aerobic process that does not favor denitrification.

Response: Thank you for pointing out the problems. Because the experiment has variability, everyone's results are not consistent, but the amount of ammonia increase in our experiment is very small, less than ten percent, and it has been changed in the manuscript.

General comment: What about the safety concerns associated with strong pathogenicity of Klebsiella pneumonia? How does this relate to practical applicability? The propagation of an antibiotic-resistant pathogen is unlikely to be approved by the relevant authorities?

Response: Thank you for the valuable suggestions you have provided. Our study is on the biodegradation of veterinary drug residues of tylosin using microbial bacteria. As you said, the problem of bacterial resistance cannot be ignored, but bacterial resistance is a prerequisite for their ability to exercise degradation. In this investigation, our main aim was to study and optimize its degradation capacity. In subsequent studies, we will focus on its degradation mechanism. In addition, screening functional genes for heterologous expression or gene knockdown validation

is also what we are studying now. Currently, we have purified proteins that can degrade tylosin and are actively studying their mechanism of action, which will be submitted in a new manuscript. The purified protein has been evaluated for safety and will be a valuable resource for the production of tylosin detergents. Thank you again for your professional and excellent comments.

Reviewer 2: The abstract needs to be re-written to reflect the significance of the study.Previous researches have reported the use of bacteria for the remediation of pollutant species. Authors should add some of these papers include their degradation efficiency

Response: Thank you very much for pointing out the problems in the manuscript.We have revised the abstract of the manuscript. We have tried our best to incorporate critical contributions of this research in the mentioned sections as per your recommendation.

I would like to thank the editor and reviewers for their valuable suggestions on our manuscript, which have led to further improvement of our article. All of your positive comments on our manuscript are appreciated with thanks. PLOS ONE is an influential journal and from all the papers published in your journal, readers have been learning a lot. We hope that now our article will be considered for publication in your esteemed journal. Should there been any other corrections we could make, please feel free to contact us.

Look forward to hearing from you back.

Regards.

Yunhang Gao
