# Supplementary material for: Development and assessment of an immobilized bacterial alliance that efficiently degrades tylosin in wastewater
Source: PLoS One. 2024 May 31;19(5):e0304113. doi: 10.1371/journal.pone.0304113 (PMC11142594; doi:10.1371/journal.pone.0304113)
Supplement: Attachment — Submitted filename: Response to Reviewers.pdf [file pone.0304113.s004.pdf]

Response Letter

Manuscript ID:PONE-D-23-42913R1

Title: Development and assessment of an immobilized bacterial alliance that efficiently degrades tylosin in wastewater

Dear Editor,

Thank you very much for giving us an opportunity to submit a revised draft of the manuscript titled “ Development and assessment of an immobilized bacterial alliance that efficiently degrades tylosin in wastewater” for publication in the PLOS ONE. We highly appreciate your good self and the reviewers for dedicating time and efforts to providing feedback on our manuscript and we are grateful for the insightful comments and valuable suggestions for the improvement of our article. Their suggestion are of course worth learning. We have incorporated all the suggestions made by the reviewers. We have made extensive corrections to our previous draft and the modified parts are marked in red in the article. The following are responses to the reviewer's comments one by one.

Response:

Thank you for pointing out these problems in manuscript. We have made changes based on your valuable comments.

Reviewer 1

Comment : Lines 82-84: You should not give description of agricultural environment in the aim of the work. It can be indicated in the introduction above.

Response: Thank you for pointing out the problems in the manuscript.This part of the manuscript has been revised.

Comment : Lines 275-278: Just define SEM in parentheses rather than in a separate sentence, it's redundant.

Response: Thank you for the valuable suggestions you have provided. Corrections have been made in the manuscript.

Comment : Line 298: pH, not PH

Response: Thank you for your comment.Changes have been made in this part of the manuscript.

Comment : Be clear about whether you are working with aquaculture or agricultural wastewater?

Response: Thank you very much for pointing out the problem in the manuscript. Sorry for the confusion caused by our misrepresentation, in the manuscript we are treating agricultural wastewater.

Comment : Lines 369-375: Do you mean that composite carbon is sourced from beads and those beads are being biodegraded? Because it seems that wastewater did not have undissolved organics.

Response: Thank you for your comment.Both TYL and immobilized carriers are organic, and both can provide a carbon source where carriers can adsorb large

amounts of pollutants,however, long-term use and complete removal are still dependent on biodegradation.

Reviewer 2

Comment : The Line 79-80 should be supported with a hypothesis to give deeper insight into the objective of the study.

Response: We would like to thank you cordially for your review and valuable suggestions for improvement of the quality of our article.We have added a discourse to this part of the manuscript based on your suggestions

I would like to thank the editor and reviewers for their valuable suggestions on our manuscript, which have led to further improvement of our article. All of your positive comments on our manuscript are appreciated with thanks. PLOS ONE is an influential journal and from all the papers published in your journal, readers have been learning a lot. We hope that now our article will be considered for publication in your esteemed journal. Should there been any other corrections we could make, please feel free to contact us.

Look forward to hearing from you back.

Regards.

Yunhang Gao
